# A Case of Monocular Hysterical Amblyopia

**Published:** 1907-06

**Authors:** D. B. St. John Roosa

**Affiliations:** Professor of Diseases of the Eye and Ear, New York Post Graduate Medical School and Hospital


### CLINICAL REPORTS.

### A Case of Monocular Hysterical Amblyopia.

By D. B. St. John Roosa, M.D.
Professor of Diseases of the Eye and Ear, New York Post Graduate Medical School and Hospital.

[*The Post-Graduate, February, 1907.*]

HELEN K., aged 18, cigar maker, came to my clinic at the Post-Graduate in November, stating that she had suddenly lost the sight of the right eye. On examination it was found that she had only quantitative perception of light in that eye, while in the other the vision was 20|20. The patient stated that she found the sight defective five days ago in the morning and that it became worse during the day until it had come to be a total loss. On inquiry as to her general condition she stated that she considered herself well except as to her sight. Two months ago her left arm and forearm and knee were numb for some days, but she had gradually recovered. The patient, a Jewess, looked well, but belonged to the nervous type.

The ophthalmoscopic examination made by Dr. MacPherson, chief of the clinic, Dr. Emerson, and myself, showed beautifully clear media, the optic papillæ glistening white, the bloodvessels and the retina entirely transparent and sound. The fundus of the eye with normal vision appeared exactly the same as that of the amblyopic eye. The patient was admitted to the hospital, the urine and blood examined with negative results. She was ordered to have the ordinary diet of the hospital, to go out in the open air on all fit days, and encouraged to believe that she would fully recover her sight. No medicine was given except a placebo.

My colleague, Professor Graeme Hammond, saw the case for me at one of his clinics and confirmed the diagnosis of hysteria. Dr. Hammond found the hysterical area, that is to say, regions of numbness in some of the extremities with exaggerated sensations around these areas. He also noted that the cornea was insensible on the ambylopic side. The sight gradually returned and she was discharged from the hospital in twenty days with vision 20|20 on each side.

It is almost impossible to get either in a clinic or a hospital an exact idea of the surroundings at the home of a patient. This is a detriment in many cases of disease where the conditions are not exclusively local, and it is especially so in affections of the nervous system. There is no substitute for the knowledge to be obtained by personal view of the surroundings in the home of the patient. Yet thorough inquiries will usually determine whether any special indications for the origin of hysteria are to be found. All the authorities agree that in these are to be found usually the causes of general hysteria, which assume various forms and vary from cases of neurasthenia, with the violent outbursts, to cases like this one here given. In this there were no hysterical seizures except the numbness of one of the legs and the amblyopia of one eye. It is probable that monocular hysterical amblyopia is rare. The amblyopia is usually in both eyes. The diagnosis is really not difficult in either case, but it is certainly easier in the monocular form, for if we find that ophthalmoscopic pictures exactly the same in the non-seeing eye as in the one with normal vision, the pupillar reflex for light the same, we are well on the road to a diagnosis of malingering or hysterical amblyopia. Fuchs[1] remarks that the diagnosis between the former conditions and those of hysteria is not always without difficulty, but as he remarks, the presence of other hysterical symptoms which existed in this case will materially aid us to a conclusion.

The necessity of the examination of the *fundus oculi* by an expert in all cases of alleged monocular hysterical amblyopia is obvious. In this case I am informed that a diagnosis of retinitis

1. Lehrbuck. 2d Edition. P. 496.

and brain tumor was made by a physician who examined the case. Whether this be true or not, it is evident that the best experience is needed for that part of the investigation of any form of amblyopia. In some cases when serious lesions exist in the cerebrum or cerebellum, the ophthalmoscopic examination gives no sign. Then again there are numerous deviations from the ideal type of healthy papillæ, bloodvessels and choroid without organic disease of the eye, the optic nerve or the brain, which only a large experience can positively decide upon. The patient whose case is above given is of a race that may be called neurotic—at least in comparison with some others. Although with a humble occupation, she was of a refined, introspective type, which, all authorities agree as being somewhat disposed to hysteria.

I am inclined to think that only a small proportion of the cases that actually occur of hysterical amblyopia affecting both eyes come to the examination of an ophthalmologist. General practitioners are so generally well informed as to hysteria in general, and aware of the frequent existence of ambylopia as one of its symptoms that such patients are not often sent to a specialist unless unusual features are presented.

